# Socioeconomic disparity in transcatheter and surgical aortic valve replacement: a population study of National Inpatient Sample from 2015 to 2020

**DOI:** 10.1038/s41598-024-62797-3

**Published:** 2024-05-23

**Authors:** Renxi Li, Deyanira J. Prastein, Brian G. Choi

**Affiliations:** The George Washington University School of Medicine and Health Sciences, 2300 I St NW, Washington, DC 20052 USA

**Keywords:** TAVR, SAVR, Socioeconomic status, Income, Aortic stenosis, Cardiology, Diseases

## Abstract

There is limited data on the effect of socioeconomic status (SES) on transcatheter (TAVR) and surgical aortic valve replacement (SAVR) outcomes for aortic stenosis (AS). This study conducted a population-based analysis to assess the influence of SES on valve replacement outcomes. Patients with AS undergoing TAVR or SAVR were identified in National Inpatient Sample from Q4 2015–2020. Multivariable logistic regressions were used to compare in-hospital outcomes between patients living in neighborhoods of income at the lowest and highest quartiles. Of 613,785 AS patients, 9.77% underwent TAVR and 10.13% had SAVR. These rates decline with lower neighborhood income levels, with TAVR/SAVR ratio also declining in lower-income areas. Excluding concomitant procedures, 58,064 patients received isolated TAVR (12,355 low-income and 15,212 high-income) and 43,694 underwent isolated SAVR (10,029 low-income and 10,811 high-income). Low-income patients, in both TAVR and SAVR, were younger but had more comorbid burden. For isolated TAVR, outcomes were similar across income groups. However, for isolated SAVR, low-income patients experienced higher in-hospital mortality (aOR = 1.44, p < 0.01), pulmonary (aOR = 1.13, p = 0.01), and renal complications (aOR = 1.14, p < 0.01). They also had more transfers, longer waits for operations, and extended hospital stays. Lower-income communities had reduced access to TAVR and SAVR, with TAVR accessibility being particularly limited. When given access to TAVR, patients from lower-income neighborhoods had mostly comparable outcomes. However, patients from low-income communities faced worse outcomes in SAVR, possibly due to delays in treatment. Ensuring equitable specialized healthcare resources including expanding TAVR access in economically disadvantaged communities is crucial.

## Introduction

Aortic stenosis (AS) is a prevalent condition, especially among the elderly. In patients aged over 75 years, the prevalence of AS is 12.4%, and severe AS requiring intervention stands at 3.4%, signifying a significant disease burden^1^. Aortic valve replacement (AVR) encompasses two treatment modalities for AS: surgical aortic valve replacement (SAVR) and transcatheter aortic valve replacement (TAVR). SAVR used to be the standard treatment modality for severe symptomatic AS^2^. In recent times, TAVR has emerged as a less invasive alternative, especially for patients deemed high-risk or unsuitable for traditional surgery^3,4^.

Disparities in healthcare outcomes are often associated with socioeconomic status (SES). A significant body of research indicates that people with lower SES tend to face increased post-operative mortality following various surgical intervention procedures^5–8^. When it comes to cardiovascular diseases, the population from lower SES typically shows a greater prevalence of risk factors. Such disparities often stem from restricted access to specialized medical care and lower adherence to prescribed treatments^5,8,9^.

In patients undergoing AVR, while prior research has found SES disparities in accessing TAVR^10–12^, there is limited data on the effect of SES on post-procedural outcomes in either TAVR or SAVR. Thus, this study aimed to conduct a population-based analysis to assess the influence of SES on AVR outcomes.

## Methods

### Data source

Using the National/Nationwide Inpatient Sample (NIS), patients diagnosed with aortic stenosis (AS) who underwent either TAVR or SAVR procedures were identified. This identification was based on the International Classification of Diseases, 10th Revision, Procedure Coding System (ICD-10-PCS) and the International Classification of Diseases, 10th Revision, Clinical Classification (ICD-10-CM) from the last quarter of 2015 to 2020. The specific codes used for AS included I06.0, I06.1, I06.2, I06.8, I06.9, Q23.1, I35.1, I35.0, and Q23.0. For TAVR, the codes were 02RF3JZ, 02RF38Z, 02RF3KZ, 02RF37Z, 02RF3JH, 02RF38H, 02RF3KH, and 02RF37H. For SAVR, the codes used were 02RF08Z, 02RF0JZ, 02RF0KZ, and 02RF07Z. The study excluded patients under the age of 18 years.

The NIS database categorizes patients into quartiles based on the estimated median household income of residents in the patient's ZIP Code. Accordingly, patients were grouped into these four quartiles reflecting their neighborhood's income. Table 1 provides a breakdown of patients from each of these income quartiles who were diagnosed with AS and underwent TAVR or SAVR. To assess the impact of SES on AVR, only isolated TAVR or SAVR were considered, excluding concomitant procedures including coronary artery bypass grafting (CABG; ICD-10-PCS: 0210xxx), percutaneous intervention (PCI; ICD-10-PCS: 02703xx, 02713xx, 02723xx, 02733xx), and mitral valve replacement (MVR; ICD-10-PCS: 02RGxxx). Patients from neighborhoods in the lowest and highest income quartiles were chosen as the cohorts for comparison in isolated TAVR and SAVR, respectively.

**Table 1:** A summary of patients from various SES backgrounds diagnosed with AS and who underwent TAVR/SAVR and isolated TAVR/SAVR. TAVR/SAVR includes isolated TAVR/SAVR and AVR with concomitant procedures.

	Neighborhood household income at 1st quartile (0–25%)	Neighborhood household income at 2nd quartile (25–50%)	Neighborhood household income at 3rd quartile (50–75%)	Neighborhood household income at 4th quartile (75–100%)	Total
AS diagnosis, n (%)	147,913 (24.1%)	160,677 (26.18%)	157,846 (25.72%)	147,349 (24.01%)	613,785 (100%)
TAVR, n (%)	12,753 (21.26%)	15,573 (25.97%)	15,941 (26.58%)	15,709 (26.19%)	59,976 (100%)
Isolated TAVR, n (%)	12,355 (21.28%)	15,067 (25.95%)	15,430 (26.57%)	15,212 (26.2%)	58,064 (100%)
Patients with AS underwent TAVR by percentage	8.62%	9.69%	10.10%	10.66%	9.77%
aOR of patients with AS underwent TAVR, 95% CI, p-value	Ref	1.03, 0.99–1.07, 0.11	1.08, 1.04–1.12, < 0.01	1.06, 1.03–1.10, < 0.01	NA
SAVR, n (%)	14,400 (23.16%)	16,478 (26.5%)	16,450 (26.46%)	14,843 (23.87%)	62,171 (100%)
Isolated SAVR, n (%)	10,029 (22.95%)	11,365 (26.01%)	11,489 (26.29%)	10,811 (24.74%)	43,694 (100%)
Patients with AS underwent SAVR by percentage	9.74%	10.26%	10.42%	10.07%	10.13%
aOR of patients with AS underwent SAVR, 95% CI, p-value	Ref	1.03, 0.99–1.06, 0.17	1.06, 1.03–1.10, < 0.01	1.14, 1.10–1.18, < 0.01	NA
Ratio of TAVR/SAVR	0.89	0.95	0.97	1.06	0.96

*AS* aortic stenosis, *AVR* aortic valve replacement, *SAVR* surgical aortic valve replacement, *NA* not applicable, *Ref* reference, *TAVR* transcatheter aortic valve replacement.

### Pre-procedural variables

Tables 2, 3, 4 and 5 summarize the comparison of pre-procedural factors for isolated TAVR and SAVR between patients from low and high-income neighborhoods. These factors encompass age, sex, primary payor status, and various hospital characteristics. Hospital characteristics include bed size, geographical location, and teaching status of the hospital. The classification of hospital bed sizes is determined by both the hospital's location and its teaching status. The American Hospital Association's annual survey refines these categories into small, medium, and large. Patient comorbidities were ascertained using the Elixhauser measure^13^ and ICD-10-CM codes, as detailed in Supplementary Table S1. In addition, All Patients Refined Diagnosis Related Groups (APR-DRG) Risk of Mortality Subclass was calculated to characterize the risk of the patients^14^.

**Table 2:** Demographic, primary payer status, hospital characteristics, transfer status, and admission status comparing patients living in neighborhoods of income at the lowest and highest quartiles who underwent isolated TAVR.

	Low SES (n = 12,355), n (%)	High SES (n = 15,212), n (%)	p-value
Sex			
Male	6500 (52.61%)	8660 (56.93%)	< 0.01
Female	5853 (47.37%)	6551 (43.06%)	< 0.01
Age			
Age < 55 years	186 (1.51%)	133 (0.87%)	< 0.01
55 ≤ Age < 65 years	752 (6.09%)	498 (3.27%)	< 0.01
65 ≤ Age < 75 years	2954 (23.91%)	2966 (19.5%)	< 0.01
75 ≤ Age < 85 years	5259 (42.57%)	6457 (42.45%)	0.84
Age ≥ 85 years	3204 (25.93%)	5158 (33.91%)	< 0.01
Race and ethnicity			
Caucasian	9630 (77.94%)	13,169 (86.57%)	< 0.01
African American	1138 (9.21%)	316 (2.08%)	< 0.01
Hispanic	884 (7.15%)	492 (3.23%)	< 0.01
Asian	77 (0.62%)	344 (2.26%)	< 0.01
Native Americans	65 (0.53%)	21 (0.14%)	< 0.01
Other races	198 (1.6%)	475 (3.12%)	< 0.01
Primary Payer status			
Medicare	10,773 (87.2%)	13,579 (89.27%)	< 0.01
Medicaid	262 (2.12%)	128 (0.84%)	< 0.01
Private insurance	950 (7.69%)	1273 (8.37%)	0.04
Self-pay	68 (0.55%)	60 (0.39%)	0.06
No charge	5 (0.04%)	3 (0.02%)	0.48
Other payment	276 (2.23%)	163 (1.07%)	< 0.01
Hospital characteristics			
Rural hospital	278 (2.25%)	26 (0.17%)	< 0.01
Urban private practice	1226 (9.92%)	1112 (7.31%)	< 0.01
Urban teaching hospital	10,851 (87.83%)	14,074 (92.52%)	< 0.01
Small bed size	1017 (8.23%)	917 (6.03%)	< 0.01
Medium bed size	2585 (20.92%)	3466 (22.78%)	< 0.01
Large bed size	8753 (70.85%)	10,829 (71.19%)	0.54
Transfer in from other hospital facilities			
No transfer in	11,494 (93.03%)	14,336 (94.24%)	< 0.01
Transferred in from a different acute care hospital	645 (5.22%)	661 (4.35%)	< 0.01
Transferred in from another type of health facility	172 (1.39%)	178 (1.17%)	0.10
Admission status			
Emergent	2120 (17.16%)	2402 (15.79%)	< 0.01
Elective	10,186 (82.77%)	12,740 (84.14%)	< 0.01

*SES* socioeconomic status, *TAVR* transcatheter aortic valve replacement.

**Table 3:** APR-DRG score, comorbidities, and clinical conditions comparing patients living in neighborhoods of income at the lowest and highest quartiles who underwent isolated TAVR.

	Low SES (n = 12,355), n (%)	High SES (n = 15,212), n (%)	p-value
APR-DRG risk of mortality subclass			
Class 1	1346 (10.89%)	1920 (12.62%)	< 0.01
Class 2	5616 (45.46%)	7120 (46.81%)	0.03
Class 3	4269 (34.55%)	4927 (32.39%)	< 0.01
Class 4	1124 (9.1%)	1245 (8.18%)	0.01
Comorbidities			
Acquired immune deficiency syndrome	11 (0.09%)	22 (0.14%)	0.22
Alcohol abuse	147 (1.19%)	194 (1.28%)	0.55
Autoimmune conditions	537 (4.35%)	764 (5.02%)	0.01
Lymphoma	100 (0.81%)	170 (1.12%)	0.01
Leukemia	87 (0.7%)	153 (1.01%)	0.01
Metastatic cancer	65 (0.53%)	123 (0.81%)	0.01
Solid tumor without metastasis, in situ	2 (0.02%)	5 (0.03%)	0.47
Solid tumor without metastasis, malignant	294 (2.38%)	464 (3.05%)	< 0.01
Cerebrovascular disease	295 (2.39%)	353 (2.32%)	0.72
Heart Failure	10 (0.08%)	6 (0.04%)	0.21
Dementia	531 (4.3%)	641 (4.21%)	0.74
Depression	915 (7.41%)	1221 (8.03%)	0.06
Diabetes without chronic complications	2156 (17.45%)	2213 (14.55%)	< 0.01
Diabetes with chronic complications	3012 (24.38%)	2924 (19.22%)	< 0.01
Drug abuse	50 (0.4%)	39 (0.26%)	0.03
Complicated hypertension	8241 (66.7%)	9617 (63.22%)	< 0.01
Uncomplicated hypertension	2816 (22.79%)	3823 (25.13%)	< 0.01
Moderate to advanced liver disease	15 (0.12%)	32 (0.21%)	0.08
Chronic pulmonary disease	3795 (30.72%)	3602 (23.68%)	< 0.01
Obesity	2763 (22.36%)	2617 (17.2%)	< 0.01
Paralysis	213 (1.72%)	217 (1.43%)	0.05
Peripheral vascular disease	2776 (22.47%)	3253 (21.38%)	0.03
Advanced renal failure	475 (3.84%)	470 (3.09%)	< 0.01
Hypothyroidism	2380 (19.26%)	3104 (20.4%)	0.02
Other thyroid disorders	133 (1.08%)	238 (1.56%)	< 0.01
Coronary artery disease	8551 (69.21%)	10,161 (66.8%)	< 0.01
Left ventricle dysfunction	28 (0.23%)	39 (0.26%)	0.71
Pulmonary hypertension	2084 (16.87%)	2334 (15.34%)	< 0.01
Endocarditis	29 (0.23%)	39 (0.26%)	0.81
Atrial fibrillation	4447 (35.99%)	5861 (38.53%)	< 0.01
Atrial flutter	409 (3.31%)	553 (3.64%)	0.15
Ventricular fibrillation	80 (0.65%)	77 (0.51%)	0.13
Ventricular flutter	2 (0.02%)	1 (0.01%)	0.59
Sick sinus syndrome	351 (2.84%)	446 (2.93%)	0.66
First-degree atrioventricular block	633 (5.12%)	1049 (6.9%)	< 0.01
Second-degree atrioventricular block	145 (1.17%)	227 (1.49%)	0.02
Complete atrioventricular block	1017 (8.23%)	1449 (9.53%)	< 0.01
Carotid artery disease	13 (0.11%)	14 (0.09%)	0.85
Hyperlipidemia	8801 (71.23%)	11,356 (74.65%)	< 0.01
Anemia	845 (6.84%)	878 (5.77%)	< 0.01
Thrombocytopenia	1161 (9.4%)	1574 (10.35%)	0.01
Sleep apnea	2059 (16.67%)	2421 (15.92%)	0.09
Tobacco use	4977 (40.28%)	6098 (40.09%)	0.75
Previous MI	1595 (12.91%)	1723 (11.33%)	< 0.01
Previous CVA	2640 (21.37%)	3368 (22.14%)	0.12
Previous CABG	2023 (16.37%)	2228 (14.65%)	< 0.01
Previous PCI	1490 (12.06%)	1750 (11.5%)	0.16
Previous valve replacement	397 (3.21%)	463 (3.04%)	0.42

*APR-DRG* All patients refined diagnosis related groups, *CABG* coronary artery bypass grafting, *CVA* cerebrovascular accident, *MI* myocardial infarction, *PCI* percutaneous coronary intervention, *SES* socioeconomic status, *TAVR* transcatheter aortic valve replacement.

**Table 4:** Demographic, primary payer status, hospital characteristics, transfer status, and admission status comparing patients living in neighborhoods of income at the lowest and highest quartiles who underwent isolated SAVR.

	Low SES (n = 10,029), n (%)	High SES (n = 10,811), n (%)	p-value
Sex			
Male	6276 (62.58%)	7307 (67.59%)	< 0.01
Female	3751 (37.4%)	3503 (32.4%)	< 0.01
Age			
Age < 55 years	2450 (24.43%)	2115 (19.56%)	< 0.01
55 ≤ Age < 65 years	2610 (26.02%)	2736 (25.31%)	0.24
65 ≤ Age < 75 years	3145 (31.36%)	3707 (34.29%)	< 0.01
75 ≤ Age < 85 years	1685 (16.8%)	2055 (19.01%)	< 0.01
Age ≥ 85 years	139 (1.39%)	198 (1.83%)	0.01
Race and ethnicity			
Caucasian	6887 (68.67%)	8608 (79.62%)	< 0.01
African American	1390 (13.86%)	323 (2.99%)	< 0.01
Hispanic	1050 (10.47%)	582 (5.38%)	< 0.01
Asian	94 (0.94%)	407 (3.76%)	< 0.01
Native Americans	77 (0.77%)	30 (0.28%)	< 0.01
Other races	208 (2.07%)	461 (4.26%)	< 0.01
Primary Payer status			
Medicare	5168 (51.53%)	5426 (50.19%)	0.05
Medicaid	1371 (13.67%)	516 (4.77%)	< 0.01
Private insurance	2804 (27.96%)	4521 (41.82%)	< 0.01
Self-pay	341 (3.4%)	137 (1.27%)	< 0.01
No charge	27 (0.27%)	19 (0.18%)	0.18
Other payment	5168 (51.53%)	5426 (50.19%)	0.05
Hospital characteristics			
Rural hospital	337 (3.36%)	11 (0.1%)	< 0.01
Urban private practice	1222 (12.18%)	988 (9.14%)	< 0.01
Urban teaching hospital	8470 (84.46%)	9812 (90.76%)	< 0.01
Small bed size	865 (8.62%)	867 (8.02%)	0.12
Medium bed size	2284 (22.77%)	2282 (21.11%)	< 0.01
Large bed size	6880 (68.6%)	7662 (70.87%)	< 0.01
Transfer in from other hospital facilities			
No transfer in	8683 (86.58%)	9780 (90.46%)	< 0.01
Transferred in from a different acute care hospital	1127 (11.24%)	876 (8.1%)	< 0.01
Transferred in from another type of health facility	186 (1.85%)	138 (1.28%)	< 0.01
Admission status			
Emergent	3258 (32.49%)	2617 (24.21%)	< 0.01
Elective	6743 (67.42%)	8159 (75.71%)	< 0.01

*SES* socioeconomic status, *SAVR* surgical aortic valve replacement.

**Table 5:** APR-DRG score, comorbidities, and clinical conditions comparing patients living in neighborhoods of income at the lowest and highest quartiles who underwent isolated SAVR.

	Low SES (n = 10,029), n (%)	High SES (n = 10,811), n (%)	p-value
APR-DRG risk of mortality subclass			
Class 1	1500 (14.96%)	1926 (17.82%)	< 0.01
Class 2	2980 (29.71%)	3571 (33.03%)	< 0.01
Class 3	3161 (31.52%)	3366 (31.13%)	0.56
Class 4	2388 (23.81%)	1948 (18.02%)	< 0.01
Comorbidities			
Acquired immune deficiency syndrome	66 (0.66%)	27 (0.25%)	< 0.01
Alcohol abuse	358 (3.57%)	292 (2.7%)	< 0.01
Autoimmune conditions	311 (3.1%)	338 (3.13%)	0.94
Lymphoma	37 (0.37%)	61 (0.56%)	0.04
Leukemia	23 (0.23%)	31 (0.29%)	0.50
Metastatic cancer	25 (0.25%)	35 (0.32%)	0.37
Solid tumor without metastasis, in situ	2 (0.02%)	2 (0.02%)	1.00
Solid tumor without metastasis, malignant	100 (1%)	128 (1.18%)	0.21
Cerebrovascular disease	199 (1.98%)	154 (1.42%)	< 0.01
Heart Failure	3 (0.03%)	6 (0.06%)	0.51
Dementia	103 (1.03%)	76 (0.7%)	0.01
Depression	986 (9.83%)	895 (8.28%)	< 0.01
Diabetes without chronic complications	1290 (12.86%)	983 (9.09%)	< 0.01
Diabetes with chronic complications	1591 (15.86%)	1343 (12.42%)	< 0.01
Drug abuse	476 (4.75%)	173 (1.6%)	< 0.01
Complicated hypertension	3645 (36.34%)	3214 (29.73%)	< 0.01
Uncomplicated hypertension	4100 (40.88%)	4770 (44.12%)	< 0.01
Moderate to advanced liver disease	10 (0.1%)	12 (0.11%)	0.83
Chronic pulmonary disease	2348 (23.41%)	1698 (15.71%)	< 0.01
Obesity	2670 (26.62%)	2369 (21.91%)	< 0.01
Paralysis	147 (1.47%)	93 (0.86%)	< 0.01
Peripheral vascular disease	2468 (24.61%)	3195 (29.55%)	< 0.01
Advanced renal failure	226 (2.25%)	117 (1.08%)	< 0.01
Hypothyroidism	1186 (11.83%)	1375 (12.72%)	0.05
Other thyroid disorders	109 (1.09%)	181 (1.67%)	< 0.01
Coronary artery disease	4016 (40.04%)	10,161 (66.8%)	< 0.01
Left ventricle dysfunction	45 (0.45%)	38 (0.35%)	0.27
Pulmonary hypertension	1458 (14.54%)	1194 (11.04%)	< 0.01
Endocarditis	1015 (10.12%)	808 (7.47%)	< 0.01
Atrial fibrillation	4108 (40.96%)	4869 (45.04%)	< 0.01
Atrial flutter	705 (7.03%)	891 (8.24%)	< 0.01
Ventricular fibrillation	234 (2.33%)	195 (1.8%)	0.01
Ventricular flutter	0 (0%)	2 (0.02%)	0.50
Sick sinus syndrome	227 (2.26%)	251 (2.32%)	0.78
First-degree atrioventricular block	327 (3.26%)	500 (4.62%)	< 0.01
Second-degree atrioventricular block	97 (0.97%)	118 (1.09%)	0.41
Complete atrioventricular block	749 (7.47%)	1449 (9.53%)	< 0.01
Carotid artery disease	8 (0.08%)	5 (0.05%)	0.41
Hyperlipidemia	5313 (52.98%)	6418 (59.37%)	< 0.01
Anemia	506 (5.05%)	286 (2.65%)	< 0.01
Thrombocytopenia	2881 (28.73%)	3604 (33.34%)	< 0.01
Sleep apnea	1512 (15.08%)	1866 (17.26%)	< 0.01
Tobacco use	4439 (44.26%)	3895 (36.03%)	< 0.01
Previous MI	569 (5.67%)	423 (3.91%)	< 0.01
Previous CVA	570 (5.68%)	640 (5.92%)	0.48
Previous CABG	377 (3.76%)	315 (2.91%)	< 0.01
Previous PCI	643 (6.41%)	642 (5.94%)	0.16
Previous valve replacement	350 (3.49%)	366 (3.39%)	0.70

*APR-DRG* All patients refined diagnosis related groups, *CABG* coronary artery bypass grafting, *CVA* cerebrovascular accident, *MI* myocardial infarction, *PCI* percutaneous coronary intervention, *SES* socioeconomic status, *SAVR* surgical aortic valve replacement.

### Outcome variables

Post-procedural outcomes encompassed in-hospital mortality, major adverse cardiovascular events (MACE), myocardial infarction (MI), stroke, transient ischemic attack (TIA), neurological and pericardial complications, pacemaker implantation, cardiogenic shock, respiratory complications, mechanical ventilation, acute kidney injury (AKI), post-procedural renal failure, venous thromboembolism (VTE), pulmonary embolism (PE), hemorrhage/hematoma, surgeries reopened for bleeding control, infections, sepsis, both deep and superficial wound complications, vascular complications, diaphragmatic paralysis, transfers to other hospital facilities, the wait time from admission to operation, hospital length of stay (LOS), and the total hospital charge. The specific ICD-10 codes used to define these outcomes are detailed in Supplementary Table S1. It is important to note that the NIS database does not capture post-discharge patient follow-up. As a result, all post-TAVR/SAVR outcomes presented in this study are derived exclusively from in-hospital data.

### Statistical analysis

Binary pre-procedural variables for patients from low- and high-income neighborhoods undergoing isolated TAVR or SAVR were assessed using Fisher's exact test. To calculate the odds of patients from different quartiles of neighborhood household income undergoing SAVR and TAVR compared to those from the lowest quartile of neighborhood household income, as well as binary post-procedural outcomes after isolated TAVR or SAVR, multivariable logistic regression was used, adjusting for pre-procedural variables that exhibited substantial differences (p < 0.1) in the Fisher's exact test. This provided adjusted odds ratios (aORs) along with their 95% confidence intervals (CI). Continuous variables, including time from admission to operation, LOS, and total hospital charges, were analyzed using generalized linear models (GLM), adjusting for all pre-procedural factors.

All statistical analyses were conducted using SAS (version 9.4). A p-value below 0.05 was considered statistically significant. The authors had full access to the dataset and assumed full responsibility for the accuracy and integrity of the analyses. Given the de-identified nature of the NIS database and the study's retrospective design, it received an exemption from the Institutional Review Board (IRB) approval at The George Washington University.

### Ethics approval

This study was exempt from the IRB approval by The George Washington University as it analyzed retrospective, deidentified NIS data.

## Results

A summary of the distribution among patients of different SES who had diagnosis of AS and those who underwent TAVR or SAVR are shown in Table 1. From the last quarter of 2015 to 2020, there were 613,785 adult patients presented with AS diagnosis, where 59,976 (9.77%) and 62,171 (10.13%) of them underwent TAVR and \SAVR, respectively. The rates that AS patients underwent TAVR or SAVR gradually decreased with lower average income in the neighborhoods, even after adjusting for pre-procedural differences as shown by the significant aORs in Table 1. In addition, the ratio of TAVR/SAVR decreased with lower income in the community. After excluding concomitant procedures, 58,064 patients underwent isolated TAVR, and 43,694 underwent isolated SAVR. In isolated TAVR, 12,355 (21.28%) patients resided in neighborhoods with incomes in the lowest quartile, while 15,212 (26.2%) lived in neighborhoods with incomes in the highest quartile. For isolated SAVR, 10,029 (22.95%) patients were from neighborhoods in the lowest income quartile, and 10,811 (24.74%) were from those in the highest income quartile.

In isolated TAVR, compared to patients living in the higher-income neighborhoods, those from lower-income neighborhoods were more likely to be female (p < 0.01), aged less than 75 years (p < 0.01), be African American (p < 0.01), Hispanic (p < 0.01), Native Americans (p < 0.01), use Medicaid (p < 0.01) as the primary payer, stay in a rural hospital (p < 0.01), urban private practice (p < 0.01), or a hospital with small bed size (p < 0.01), transferred in from a different acute care hospital (p < 0.01), and under emergent admission (p < 0.01) (Table 2). In contrast, patients from lower-income neighborhoods were less likely to have age over 85 years (p < 0.01), be Caucasian (p < 0.01) or Asian (p < 0.01), use Medicare (p < 0.01) or private insurance (p = 0.04) as the primary payer, or stay in urban teaching hospitals (p < 0.01) (Table 2).

In isolated TAVR, patients from lower-income neighborhoods were more likely to have APR-DRG Class 3 (p < 0.01) and Class 4 (p = 0.01), diabetes (p < 0.01), drug abuse (p = 0.03), complicated hypertension (p < 0.01), chronic pulmonary disease (p < 0.01), obesity (p < 0.01), peripheral vascular disease (p = 0.03), advanced renal failure (p < 0.01), pulmonary hypertension (p < 0.01), anemia (p < 0.01), coronary artery disease (p < 0.01), previous myocardial infarction (MI; p < 0.01) and previous CABG (p < 0.01) (Table 3). In contrast, patients from lower-income neighborhoods were less likely to have autoimmune conditions (p = 0.01), lymphoma (p = 0.01), leukemia (p = 0.01), metastatic cancer (p = 0.01), solid tumor without metastasis, malignant (p < 0.01), uncomplicated hypertension (p < 0.01), hypothyroidism (p = 0.02), other thyroid disorders (p < 0.01), atrial fibrillation (p < 0.01), first-degree (p < 0.01), second-degree (p = 0.02), or complete atrioventricular block (p < 0.01), hyperlipidemia (p < 0.01), or thrombocytopenia (p = 0.01) (Table 3).

In isolated SAVR, patients from lower-income neighborhoods were more likely to be female (p < 0.01), aged less than 55 years (p < 0.01), be African American (p < 0.01), Hispanic (p < 0.01), Native American (p < 0.01), use Medicaid (p < 0.01) and self-pay (p < 0.01) as the primary payer, stay in rural hospital (p < 0.01), urban private practice (p < 0.01), hospitals with medium bed size (p < 0.01), transferred in from a different acute care hospital (p < 0.01), transferred in from another type of health facility (p < 0.01), and under emergent presentation (p < 0.01) (Table 4). Patients from lower-income neighborhoods were less likely to have ages over 65 years (p < 0.01), be Caucasian (p < 0.01) or Asian (p < 0.01), under private insurance (p < 0.01), stay in urban teaching hospital (p < 0.01) or a hospital with large bed size (p < 0.01) (Table 4).

In isolated SAVR, compared to patients from high-income neighborhoods, those living in lower-income neighborhoods were more likely to have APR-DRG class 4 (p < 0.01), acquired immune deficiency syndrome (p < 0.01), alcohol abuse (p < 0.01), cerebrovascular disease (p < 0.01), dementia (p = 0.01), depression (p < 0.01), diabetes (p < 0.01), drug abuse (p < 0.01), complicated hypertension (p < 0.01), chronic pulmonary disease (p < 0.01), obesity (p < 0.01), paralysis (p < 0.01), advanced renal failure (p < 0.01), pulmonary hypertension (p < 0.01), endocarditis (p < 0.01), ventricular fibrillation (p = 0.01), anemia (p < 0.01), tobacco use (p < 0.01), previous MI (p < 0.01), previous CABG (p < 0.01) (Table 5). On the other hand, patients from lower-income neighborhoods were less likely to have lymphoma (p = 0.04), uncomplicated hypertension (p < 0.01), peripheral vascular disease (p < 0.01), other thyroid disorders (p < 0.01), coronary artery disease (p < 0.01), atrial fibrillation (p < 0.01), atrial flutter (p < 0.01), first-degree (p < 0.01) or complete atrioventricular block (p < 0.01), hyperlipidemia (p < 0.01), thrombocytopenia (p < 0.01), or sleep apnea (p < 0.01) (Table 5).

**Figure 1:**
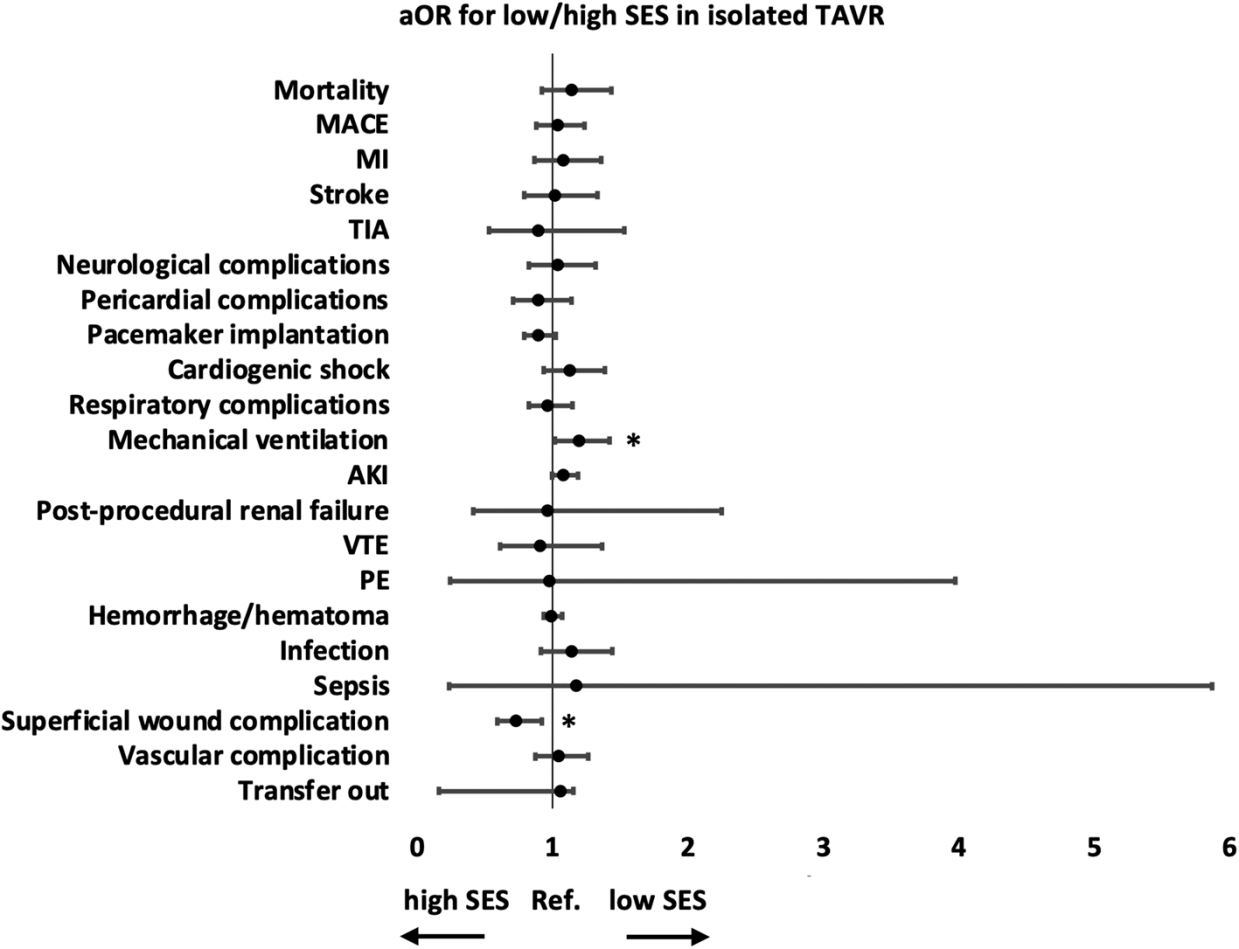
In-hospital outcomes comparing patients living in neighborhoods of income at the lowest and highest quartiles who underwent isolated TAVR. The aOR and 95% CI are shown. *p-value < 0.05. *AKI* acute kidney injury, *aOR* adjusted odds ratio, *CI* confidence interval, *MACE* major adverse cardiovascular event, *MI* myocardial infarction, *PE* pulmonary embolism, *Ref* reference, *SES* socioeconomic status, *TAVR* transcatheter aortic valve replacement, *TIA* transient ischemic attack, *VTE* venous thromboembolism.

In isolated TAVR, compared to patients living in neighborhoods of income at the highest quartiles, those living in lower-income neighborhoods had higher risks of mechanical ventilation (aOR 1.20, 95 CI 1.02–1.42, p = 0.03) but lower risks of superficial wound complication (aOR 0.74, 95 CI 0.59–0.92, p = 0.01) (Table 6 and Fig. 1). Patients from low-income neighborhoods had lower total hospital charges (207,314 ± 1106 vs 227,423 ± 1188 dollars, p < 0.01) (Table 6).

**Table 6:** In-hospital outcomes comparing patients living in neighborhoods of income at the lowest and highest quartiles who underwent isolated TAVR.

	Low SES (n = 12,355), n (%)	High SES (n = 15,212), n (%)	aOR for low/high SES (95% CI)	p-value
Mortality	175 (1.42%)	189 (1.24%)	1.14 (0.92–1.43)	0.22
MACE	277 (2.24%)	319 (2.1%)	1.04 (0.88–1.23)	0.65
MI	162 (1.31%)	179 (1.18%)	1.08 (0.86–1.36)	0.49
Stroke	104 (0.84%)	128 (0.84%)	1.02 (0.79–1.33)	0.86
TIA	25 (0.2%)	32 (0.21%)	0.90 (0.53–1.53)	0.70
Neurological complications	134 (1.08%)	163 (1.07%)	1.04 (0.83–1.32)	0.72
Pericardial complications	120 (0.97%)	174 (1.14%)	0.90 (0.71–1.14)	0.38
Pacemaker implantation	857 (6.94%)	1277 (8.39%)	0.90 (0.79–1.02)	0.10
Cardiogenic shock	235 (1.9%)	226 (1.49%)	1.14 (0.93–1.39)	0.21
Respiratory complications	287 (2.32%)	319 (2.1%)	0.97 (0.82–1.15)	0.72
Mechanical ventilation	339 (2.74%)	311 (2.04%)	1.20 (1.02–1.42)	0.03
AKI	1275 (10.32%)	1369 (9%)	1.09 (0.99–1.19)	0.07
Post-procedural renal failure	10 (0.08%)	13 (0.09%)	0.97 (0.42–2.25)	0.94
VTE	42 (0.34%)	56 (0.37%)	0.91 (0.61–1.36)	0.65
PE	4 (0.03%)	4 (0.03%)	0.98 (0.24–3.97)	0.98
Hemorrhage/hematoma	2025 (16.39%)	2422 (15.92%)	1.00 (0.93–1.07)	0.92
Infection	156 (1.26%)	160 (1.05%)	1.15 (0.91–1.44)	0.25
Sepsis	3 (0.02%)	3 (0.02%)	1.18 (0.24–5.87)	0.84
Superficial wound complication	132 (1.07%)	223 (1.47%)	0.74 (0.59–0.92)	0.01
Vascular complication	239 (1.93%)	246 (1.62%)	1.05 (0.87–1.26)	0.62
Transfer out	1376 (11.14%)	1675 (11.01%)	1.06 (0.98–1.15)	0.16

	Mean ± SE	Mean ± SE	F score	p-value
Admission to operation (days)	0.99 ± 0.03	0.89 ± 0.02	0.18	0.67
LOS (days)	3.86 ± 0.05	3.68 ± 0.40	0.24	0.62
Total hospital charge (US dollars)	207,314 ± 1106	227,423 ± 1188	173.83	< 0.01

*AKI* acute kidney injury, *aOR* adjusted odds ratio, *CI* confidence interval, *LOS* length of stay, *MACE* major adverse cardiovascular event, *MI* myocardial infarction, *NA* not applicable, *PE* pulmonary embolism, *SES* socioeconomic status, *TAVR* transcatheter aortic valve replacement, *TIA* transient ischemic attack, *VTE* venous thromboembolism.

**Figure 2:**
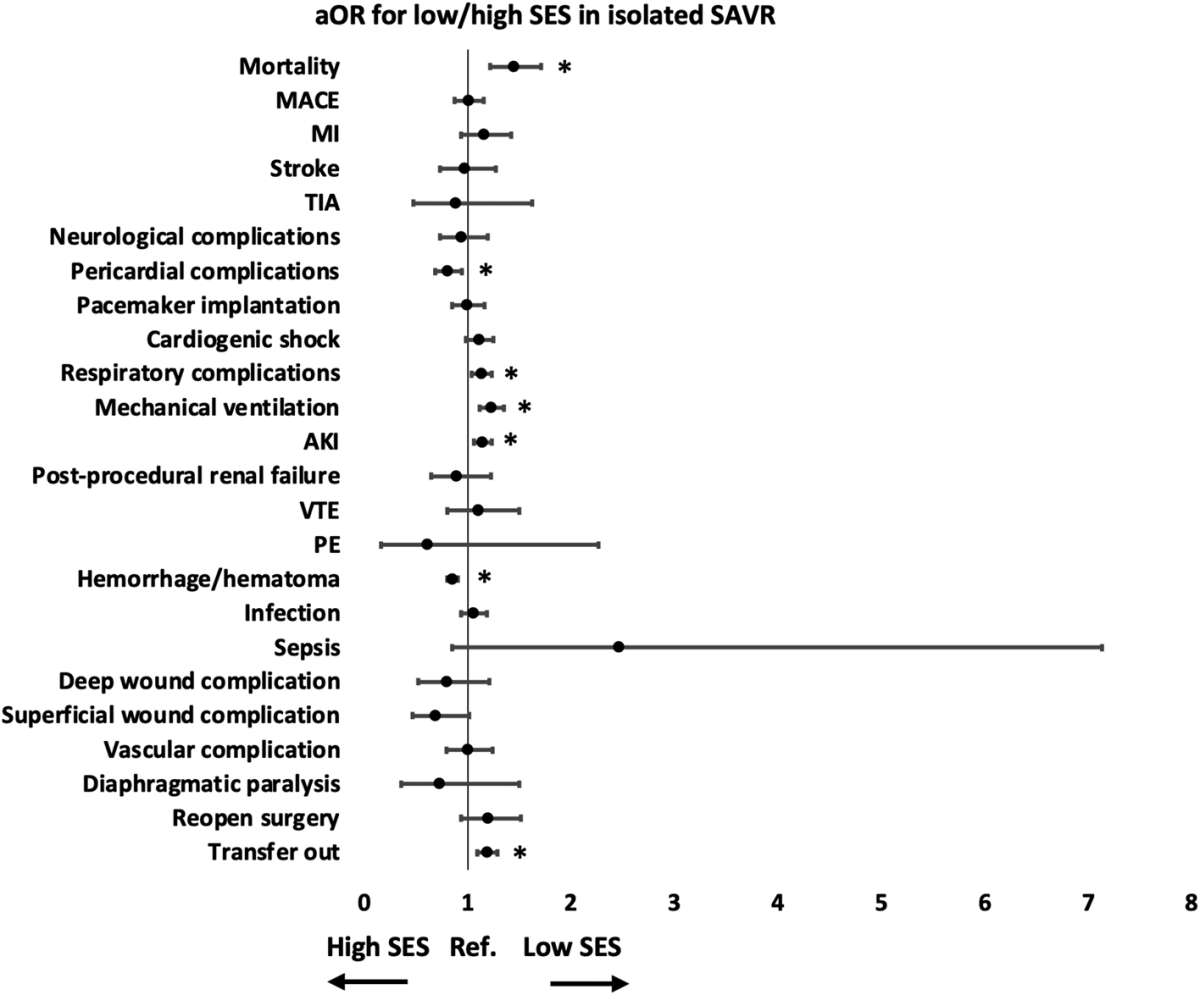
In-hospital outcomes comparing patients living in neighborhoods of income at the lowest and highest quartiles who underwent isolated SAVR. The aOR and 95 CI are shown. *p-value < 0.05. *AKI* acute kidney injury, *aOR* adjusted odds ratio, *CI* confidence interval, *MACE* major adverse cardiovascular event, *MI* myocardial infarction, *PE* pulmonary embolism, *Ref* reference, *SES* socioeconomic status, *SAVR* surgical aortic valve replacement, *TIA* transient ischemic attack, *VTE* venous thromboembolism.

In isolated SAVR, compared to patients living in neighborhoods of income at the highest quartiles, those living in lower-income neighborhoods had higher risks of mortality (aOR 1.44, 95% CI 1.22–1.71, p < 0.01), respiratory complications (aOR 1.13, 95% CI 1.03–1.23, p = 0.01), mechanical ventilation (aOR 1.23, 95% CI 1.11–1.35, p < 0.01), AKI (aOR 1.14, 95% CI 1.06–1.23, p < 0.01), and transfer out to other facilities (aOR 1.19, 95% CI 1.09–1.29, p < 0.01) (Table 7 and Fig. 2). On the other hand, patients living in lower-income neighborhoods had lower risks of pericardial complications (aOR 0.80, 95% CI 0.68–0.94, p = 0.01), and hemorrhage/hematoma (aOR 0.85, 95% CI 0.80–0.90, p < 0.01) (Table 7 and Fig. 2). Patients from low-income neighborhoods had longer time from admission to operation (2.22 ± 0.05 vs 1.42 ± 0.05 days, p = 0.01), longer LOS (11.37 ± 0.12 vs 9.39 ± 0.09, p < 0.01), but lower total hospital charge (275,909 ± 2731 vs 276,058 ± 2942 dollars, p < 0.01) (Table 7).

**Table 7:** In-hospital outcomes comparing patients living in neighborhoods of income at the lowest and highest quartiles who underwent isolated SAVR.

	Low SES (n = 10,029), n (%)	High SES (n = 10,811), n (%)	aOR for low/high SES (95% CI)	p-value
Mortality	385 (3.84%)	254 (2.35%)	1.44 (1.22–1.71)	< 0.01
MACE	446 (4.45%)	454 (4.2%)	1.00 (0.87–1.15)	0.99
MI	229 (2.28%)	187 (1.73%)	1.15 (0.93–1.42)	0.19
Stroke	108 (1.08%)	114 (1.05%)	0.97 (0.73–1.27)	0.80
TIA	18 (0.18%)	25 (0.23%)	0.88 (0.48–1.62)	0.68
Neurological complications	133 (1.33%)	145 (1.34%)	0.93 (0.73–1.19)	0.59
Pericardial complications	330 (3.29%)	377 (3.49%)	0.80 (0.69–0.94)	0.01
Pacemaker implantation	613 (6.11%)	670 (6.2%)	0.99 (0.85–1.16)	0.90
Cardiogenic shock	756 (7.54%)	645 (5.97%)	1.11 (0.98–1.24)	0.09
Respiratory complications	1311 (13.07%)	1216 (11.25%)	1.13 (1.03–1.23)	0.01
Mechanical ventilation	1263 (12.59%)	960 (8.88%)	1.23 (1.11–1.35)	< 0.01
AKI	2313 (23.06%)	1990 (18.41%)	1.14 (1.06–1.23)	< 0.01
Post-procedural renal failure	69 (0.69%)	88 (0.81%)	0.89 (0.64–1.22)	0.46
VTE	91 (0.91%)	79 (0.73%)	1.10 (0.80–1.50)	0.56
PE	4 (0.04%)	6 (0.06%)	0.61 (0.16–2.26)	0.46
Hemorrhage/hematoma	6029 (60.12%)	6915 (63.96%)	0.85 (0.80–0.90)	< 0.01
Infection	785 (7.83%)	740 (6.84%)	1.05 (0.94–1.19)	0.38
Sepsis	11 (0.11%)	5 (0.05%)	2.46 (0.85–7.13)	0.10
Deep wound complication	41 (0.41%)	49 (0.45%)	0.79 (0.52–1.21)	0.28
Superficial wound complication	53 (0.53%)	66 (0.61%)	0.69 (0.47–1.01)	0.06
Vascular complication	170 (1.7%)	174 (1.61%)	0.99 (0.80–1.24)	0.95
Diaphragmatic paralysis	12 (0.12%)	19 (0.18%)	0.73 (0.35–1.50)	0.39
Reopen surgery	146 (1.46%)	143 (1.32%)	1.19 (0.94–1.51)	0.16
Transfer out	2252 (22.45%)	1832 (16.95%)	1.19 (1.09–1.29)	< 0.01

	Mean ± SE	Mean ± SE	F score	p-value
Admission to operation (days)	2.22 ± 0.05	1.42 ± 0.05	8.58	0.01
LOS (days)	11.37 ± 0.12	9.39 ± 0.09	24.90	< 0.01
Total hospital charge (US dollars)	275,909 ± 2,731	276,058 ± 2942	23.46	< 0.01

*AKI* acute kidney injury, *aOR* adjusted odds ratio, *CI* confidence interval, *LOS* length of stay, *MACE* major adverse cardiovascular event, *MI* myocardial infarction, *NA* not applicable, *PE* pulmonary embolism, *SES* socioeconomic status, *SAVR* surgical aortic valve replacement, *TIA* transient ischemic attack, *VTE* venous thromboembolism.

## Discussion

This study conducted a population-based analysis to examine the impact of SES on post-procedural outcomes following AVR. Patients with AS from lower-income communities had less access to both TAVR and SAVR, with TAVR accessibility being particularly limited. In isolated TAVR, outcomes were largely similar between patients from both lower- and higher-income neighborhoods. However, in isolated SAVR, patients from lower-income communities had a higher risk of in-hospital mortality, as well as increased pulmonary and renal complications; additionally, these patients experienced longer waits from admission to operation and had extended hospital stays.

Although the NIS categorizes patients' SES based on their neighborhood's household income rather than the income of the patients, evidence from the population differences between the low- and high-income patients in our study validates this stratification. For example, patients from lower-income neighborhoods were more likely to be under Medicaid, a program for those with low income. Additionally, racial minorities such as African Americans, Hispanics, and Native Americans, as well as comorbidities including obesity, diabetes, uncontrolled hypertension, peripheral vascular disease, chronic pulmonary disease, severe renal failure, tobacco use, and substance abuse are all strongly associated with low SES^15–23^. These factors were more prevalent in the low-income cohort of our study, which supports the NIS's approach to stratifying SES.

The prevalence of AS rises exponentially with age^1^. Despite being at a younger age, patients from lower-income neighborhoods were still prominently represented in AS diagnoses when compared to those from higher-income areas in both TAVR and SAVR. This discrepancy might be attributed to the higher prevalence of AS risk factors in lower-income communities. Established risk factors for AS, such as obesity, diabetes, uncontrolled hypertension, and chronic kidney disease^24^, were more common among patients from lower-income neighborhoods, which may lead to their earlier onset of AS.

The rates of patients with AS undergoing either TAVR or SAVR declined with decreasing income of the community, even when accounting for pre-procedural differences including age and comorbidities. Notably, the TAVR to SAVR ratio also declined with reduced income in the neighborhood. This indicates that patients with AS from lower-income communities face barriers to accessing both TAVR and SAVR, with TAVR being especially less accessible. This aligns with prior studies that highlighted lower SES patients encounter challenges in accessing TAVR^10–12^. Such disparities may stem from the high costs associated with TAVR valves combined with inadequate Medicare reimbursements for TAVR, discouraging some hospitals from offering the procedure, which disproportionately impacts socioeconomically disadvantaged patients^12^. Consequently, during TAVR's initial growth phase in the US, hospitals catering to more affluent patients were quicker to adopt the procedure, which resulted in unequal distribution of TAVR services^10^.

In isolated TAVR, post-procedural outcomes were largely comparable between patients from both lower- and higher-income neighborhoods, aligning with findings from Rogers et al.^25^. This suggests that when provided with access, patients from lower-income communities can benefit from the novel, lifesaving TAVR as much as their counterparts in wealthier communities. Therefore, efforts should be concentrated on ensuring equitable access to TAVR for patients in economically disadvantaged communities.

In isolated SAVR, patients from lower-income neighborhoods experienced a heightened risk of in-hospital mortality and elevated pulmonary and renal complications. This contrasts with the findings by Rogers et al., who reported no significant disparities in SAVR outcomes^25^. The discrepancy might arise from the fact that Rogers et al. sourced their data solely from Florida and Washington state registries, which may not be wholly representative of the broader US population^25^. In contrast, our study utilized a comprehensive national registry. The worse SAVR outcomes in lower-income communities could be attributed to delayed diagnosis and subsequent treatment. This is underscored by the extended wait time from admission to operation in these patients, averaging 2.22 days, compared to just 1.42 days in patients from wealthier neighborhoods especially considering about one-third of SAVR cases were emergent. This delay remained significant even after accounting for pre-operative differences, such as higher comorbidity burden in patients from lower-income communities, which often necessitates additional stabilization time prior to surgery. Additionally, patients from lower-income communities were more likely to get transferred in from other facilities, leading to potentially more delays in the transfer process prior to admission. These delays may arise from limited healthcare resources in lower-income communities. Efforts should be directed towards ensuring equitable access to specialized care in these areas. Another potential factor could be the restricted access to TAVR in the low SES communities. Younger patients with AS are often directed towards SAVR, primarily because of the possibility of implanting a mechanical valve, which can reduce the need for future interventions. In contrast, older patients were typically recommended for TAVR, given the potential risks associated with open surgery and diminished necessity for long-term re-interventions. Among patients who are aged 85 and above, patients from a low SES background were less likely to be treated with TAVR (25.93% vs 33.91%, p < 0.01) compared to those of high SES. As a result, patients who might have benefited more from TAVR were disproportionately directed, leading to poorer outcomes. In light of this finding, broadening TAVR accessibility in economically disadvantaged areas becomes imperative.

This study comes with several limitations. First, while the NIS database's income stratification finds support in our findings, using neighborhood income as a proxy for individual income introduces inherent variability. However, individual income data of the patients is rarely gathered during medical visits. This makes recording exact patient income, especially in expansive national registries, a challenge, rendering neighborhood income a practical alternative. Second, the NIS database is primarily structured based on diagnostic codes. This means it does not capture granular details such as medication, and laboratory results, or allows the calculation of surgical risk scores like the STS (Society of Thoracic Surgeons) or EuroSCORE (European System for Cardiac Operative Risk Evaluation) scores. Additionally, the database only has records of the hospital stay, lacking post-discharge follow-up data. This limitation restricts our ability to evaluate long-term prognoses post-TAVR and SAVR, where previous studies indicated disadvantaged SES may negatively influence long-term outcomes in AVR^12^. Nevertheless, NIS is the most extensive in-patient database in the US that records 20% of all discharges. This breadth provides one of the most representative samples of the US patient demographic, enabling a thorough population-level examination of the impact of SES on AVR outcomes.

In this population-based analysis, we explored the influence of SES on short-term outcomes post-TAVR and SAVR. It was found that lower-income communities had reduced access to both TAVR and SAVR, with the disparity in accessibility being more pronounced for TAVR. When given access to isolated TAVR, patients from lower-income neighborhoods had mostly comparable outcomes as those from higher-income communities. However, for isolated SAVR, patients from low-income communities faced a higher risk of in-hospital mortality and increased pulmonary and renal complications, most likely due to delays in treatment. Addressing these disparities is crucial, with a particular focus on ensuring equitable specialized healthcare resources including expanding TAVR access in economically disadvantaged communities.

### Supplementary Information

Supplementary Table S1.

## Data Availability

The data that support the findings of this study are available on request from the corresponding author.
