# Supplementary material for: Socioeconomic disparity in transcatheter and surgical aortic valve replacement: a population study of National Inpatient Sample from 2015 to 2020
Source: Sci Rep. 2024 May 23;14:11762. doi: 10.1038/s41598-024-62797-3 (PMC11116551; doi:10.1038/s41598-024-62797-3)
Supplement: Supplementary file 1 — Supplementary Table S1. [file 41598_2024_62797_MOESM1_ESM.docx]

**Table S1.** The International Classification of Diseases, Tenth Revision, Clinical Modification (ICD-10-CM) and International Classification of Diseases, 10th Revision, Procedure Coding System (ICD-10-PCS) codes for comorbidities and relevant diagnosis.

| **Comorbidities and Diagnosis** | **ICD-10-CM and ICD-10-PCS** |
| --- | --- |
| **Acquired immune deficiency syndrome** | Elixhauser comorbidity |
| **Alcohol abuse** | Elixhauser comorbidity |
| **Autoimmune conditions** | Elixhauser comorbidity |
| **Lymphoma** | Elixhauser comorbidity |
| **Leukemia** | Elixhauser comorbidity |
| **Metastatic cancer** | Elixhauser comorbidity |
| **Solid tumor without metastasis, in situ** | Elixhauser comorbidity |
| **Solid tumor without metastasis, malignant** | Elixhauser comorbidity |
| **Cerebrovascular disease** | Elixhauser comorbidity |
| **Heart failure** | Elixhauser comorbidity |
| **Dementia** | Elixhauser comorbidity |
| **Depression** | Elixhauser comorbidity |
| **Diabetes without chronic complications** | Elixhauser comorbidity |
| **Diabetes with chronic complications** | Elixhauser comorbidity |
| **Drug abuse** | Elixhauser comorbidity |
| **Complicated hypertension** | Elixhauser comorbidity |
| **Uncomplicated hypertension** | Elixhauser comorbidity |
| **Moderate to advanced liver disease** | Elixhauser comorbidity |
| **Chronic pulmonary disease** | Elixhauser comorbidity |
| **Obesity** | Elixhauser comorbidity |
| **Paralysis** | Elixhauser comorbidity |
| **Peripheral vascular disease** | Elixhauser comorbidity |
| **Advanced renal failure** | Elixhauser comorbidity |
| **Hypothyroidism** | Elixhauser comorbidity |
| **Other thyroid disorders** | Elixhauser comorbidity |
| **Valvular diseases** | Elixhauser comorbidity |
| **Coronary artery disease** | I25 |
| **Left ventricle dysfunction** | I50.1 |
| **Pulmonary hypertension** | I27.2 |
| **Endocarditis** | I33, I38, I39 |
| **Atrial fibrillation** | I48.0, I48.1, I48.2, I48.91 |
| **Atrial flutter** | I48.3, I48.4, I48.92 |
| **Ventricular fibrillation** | I49.01 |
| **Ventricular flutter** | I49.02 |
| **Sick sinus syndrome** | I49.5 |
| **First-degree atrioventricular block** | I44.0 |
| **Second-degree atrioventricular block** | I44.1 |
| **Complete atrioventricular block** | I44.2 |
| **Carotid artery disease** | I65.21, I65.22, I65.23, I65.29 |
| **Hyperlipidemia** | E78.0, E78.1, E78.2, E78.3, E78.5, E78.5 |
| **Anemia** | D63 |
| **Thrombocytopenia** | D69.5, D69.6 |
| **Sleep apnea** | G47.3 |
| **Tobacco use** | F17, Z72.0, Z87.891 |
| **Previous myocardial infarction** | I25.2 |
| **Previous cerebrovascular accident** | Z86.73 |
| **Previous coronary artery bypass grafting** | Z95.1 |
| **Previous percutaneous coronary intervention** | Z98.61, Z95.5 |
| **Previous valve replacement** | Z95.2, Z95.,3 Z95.4 |

**Table S2.** The International Classification of Diseases, Tenth Revision, Clinical Modification (ICD-10-CM) and International Classification of Diseases, 10th Revision, Procedure Coding System (ICD-10-PCS) codes for perioperative outcomes.

| **Perioperative outcomes** | **ICD-10-CM and ICD-10-PCS** |
| --- | --- |
| **MACE** |  |
| Myocardial infarction (MI) | I97.710, I97.711, I97.120, I97.121, I46.9 |
| Stroke | I60.9, I61.9, I63.22, I63.139, I63.239, I63.019, I63.119, I63.219, [I97.811](https://www.icd10data.com/ICD10CM/Codes/I00-I99/I95-I99/I97-/I97.811), I97.820, [I97.821](https://www.icd10data.com/ICD10CM/Codes/I00-I99/I95-I99/I97-/I97.821), I97.810 |
| Postprocedural cardiogenic shock | T81.11XA |
| Postprocedural heart failure | I97.130, I97.131 |
| Postprocedural cardiac insufficiency | I97.110, I97.111 |
| **MI** | I97.710, I97.711, I97.120, I97.121, I46.9 |
| **Stroke** | I60.9, I61.9, I63.22, I63.139, I63.239, I63.019, I63.119, I63.219, [I97.811](https://www.icd10data.com/ICD10CM/Codes/I00-I99/I95-I99/I97-/I97.811), I97.820, [I97.821](https://www.icd10data.com/ICD10CM/Codes/I00-I99/I95-I99/I97-/I97.821), I97.810 |
| **Transient ischemic attack (TIA)** | G45.9, I67.848 |
| **Neurological complications** |  |
| Nervous system complication, unspecified | [G97.81](https://www.icd10data.com/ICD10CM/Codes/G00-G99/G89-G99/G97-/G97.81) |
| Central nervous system complication | G97.81, G97.82 |
| Iatrogenic cerebrovascular infarction or hemorrhage | [I97.811](https://www.icd10data.com/ICD10CM/Codes/I00-I99/I95-I99/I97-/I97.811), [I97.821](https://www.icd10data.com/ICD10CM/Codes/I00-I99/I95-I99/I97-/I97.821)  I97.810 |
| Transient ischemic attack | [G45.9](https://www.icd10data.com/ICD10CM/Codes/G00-G99/G40-G47/G45-/G45.9), [I67.848](https://www.icd10data.com/ICD10CM/Codes/I00-I99/I60-I69/I67-/I67.848) |
| Any stroke | 160.9, 161.9, 163.22, 163.139, 163.239  163.019, 163.119, 163.219 |
| **Pericardial complications** |  |
| Hemopericardium | I31.2 |
| Tamponade | [I31.4](https://www.icd10data.com/ICD10CM/Codes/I00-I99/I30-I52/I31-/I31.4) |
| Pericardiocentesis | 0W9DXXX, 0W9CXXX, 0W9D40Z |
| Acute pericarditis | I30.1, I30.8, I30.9 |
| **Pacemaker implantation** | 0JH606Z, 0JH636Z, 0JH806Z, 0JH836Z, 0JH60PZ, 0JH63PZ, 0JH80PZ, 0JH83PZ, 0JH604Z, 0JH634Z, 0JH804Z, 0JH834Z, 0JH605Z, 0JH635Z, 0JH805Z, 0JH835Z, 02H73KZ, 02HK3KZ, 02HL3KZ, 02HN0KZ, 02HN4KZ, 0JH608Z, 0JH638Z, 0JH808Z, 0JH838Z, 02H60KZ, 02H63KZ, 02H64KZ, 02H70KZ, 02H73KZ, 02H74KZ, 02HK0KZ, 02HK3KZ, 02HK4KZ, 02HL0KZ, 02HL3KZ, 02HL4KZ, 0JH608Z, 0JH638Z, 0JH808Z, 0JH838Z, 02H60KZ, 02H63KZ, 02H64KZ, 02H70KZ, 02H73KZ, 02H74KZ, 02HK0KZ, 02HK3KZ, 02HK4KZ, 02HL0KZ, 02HL3KZ, 02HL4KZ, 0JH608Z, 0JH638Z, 0JH808Z, 0JH838Z, |
| **Cardiogenic shock** | R57.0 |
| **Respiratory complications** |  |
| Pneumothorax/hemothorax | [J95.811](https://www.icd10data.com/ICD10CM/Codes/J00-J99/J95-J95/J95-/J95.811), [J95.812](https://www.icd10data.com/ICD10CM/Codes/J00-J99/J95-J95/J95-/J95.812), J95.830, J95.831, J94.2 |
| Diaphragm paralysis | [J98.6](https://www.icd10data.com/ICD10CM/Codes/J00-J99/J96-J99/J98-/J98.6) |
| Post-operative Respiratory Failure | [J95.821](https://www.icd10data.com/ICD10CM/Codes/J00-J99/J95-J95/J95-/J95.821), [J96.00](https://www.icd10data.com/ICD10CM/Codes/J00-J99/J96-J99/J96-/J96.00), [J95.822](https://www.icd10data.com/ICD10CM/Codes/J00-J99/J95-J95/J95-/J95.822), [J96.20](https://www.icd10data.com/ICD10CM/Codes/J00-J99/J96-J99/J96-/J96.20) |
| Pulmonary insufficiency | J95.2, J95.3 |
| Respiratory arrest | R09.2 |
| Other iatrogenic Respiratory Complications | J95.88, J95.89, J95.850, J95.851, J95.859 |
| **Mechanical ventilation** | 5A1935Z, 5A1945Z, 5A1955Z |
| **Acute kidney injury (AKI)** | N17.9, N17.0, N17.1, N17.2 |
| **Post-procedural renal failure** | N99.0 |
| **Venous thromboembolism (VTE)** | I82.401, I82.402, I82.403, I82.409, I82.411, I82.412, I82.413, I82.419, I82.421, I82.422, I82.423, I82.429, I82.431, I82.432, I82.433, I82.439, I82.441, I82.442, I82.443, I82.449, I82.451, I82.452, I82.453, I82.459, I82.461, I82.462, I82.463, I82.469, I82.491, I82.492, I82.493, I82.499, I82.4Y1, I82.4Y2, I82.4Y3, I82.4Y9, I82.4Z1, I82.4Z2, I82.4Z3, I82.4Z9 |
| **Pulmonary embolism (PE)** | I26.02, I26.09, I26.92, I26.93, I26.94 |
| **Hemorrhage/hematoma** |  |
| Hemorrhage/hematoma complicating a procedure | [I97.411](https://www.icd10data.com/ICD10CM/Codes/I00-I99/I95-I99/I97-/I97.411), [I97.418](https://www.icd10data.com/ICD10CM/Codes/I00-I99/I95-I99/I97-/I97.418), [I97.42](https://www.icd10data.com/ICD10CM/Codes/I00-I99/I95-I99/I97-/I97.42), [I97.611](https://www.icd10data.com/ICD10CM/Codes/I00-I99/I95-I99/I97-/I97.611)  [I97.618](https://www.icd10data.com/ICD10CM/Codes/I00-I99/I95-I99/I97-/I97.618), [I97.620](https://www.icd10data.com/ICD10CM/Codes/I00-I99/I95-I99/I97-/I97.620), [I97.411](https://www.icd10data.com/ICD10CM/Codes/I00-I99/I95-I99/I97-/I97.411), [I97.418](https://www.icd10data.com/ICD10CM/Codes/I00-I99/I95-I99/I97-/I97.418), [I97.42](https://www.icd10data.com/ICD10CM/Codes/I00-I99/I95-I99/I97-/I97.42), [I97.621](https://www.icd10data.com/ICD10CM/Codes/I00-I99/I95-I99/I97-/I97.621), [I97.631](https://www.icd10data.com/ICD10CM/Codes/I00-I99/I95-I99/I97-/I97.631), [I97.638](https://www.icd10data.com/ICD10CM/Codes/I00-I99/I95-I99/I97-/I97.638) |
| Acute post-hemorrhagic anemia | D62 |
| Hemorrhage requiring transfusion | 3023XXX, 3024XXX |
| **Infection** |  |
| Fever | T82.6, T82.7, R50.82 |
| Septicemia | A41.9, A65.20, [T81.12XA](https://icd.codes/icd10cm/T8112XA) |
| Post-procedural aspiration pneumonia | J95.89 |
| **Sepsis** | T81.12XA, T81.44XA |
| **Deep wound complication** | T81.32XA, T81.43XA |
| **Superficial wound complications** | L76.34, L76.32, T81.31XA, T81.41XA, T81.42XA, T81.40XA |
| **Vascular complication** |  |
| Accidental puncture or laceration during a procedure | [I97.51](https://www.icd10data.com/ICD10CM/Codes/I00-I99/I95-I99/I97-/I97.51), [I97.52](https://www.icd10data.com/ICD10CM/Codes/I00-I99/I95-I99/I97-/I97.52) |
| Injury to blood vessels | S25.X, S35.X |
| Arteriovenous Fistula | I77.0 |
| Injury to retroperitoneum | [S36.899A](https://www.icd10data.com/ICD10CM/Codes/S00-T88/S30-S39/S36-/S36.899A) |
| Vascular complication requiring surgical/percutaneous repair | [03QY0ZZ](https://www.icd10data.com/ICD10PCS/Codes/0/3/Q/Y/03QY0ZZ), [03QY3ZZ](https://www.icd10data.com/ICD10PCS/Codes/0/3/Q/Y/03QY3ZZ), [03QY4ZZ](https://www.icd10data.com/ICD10PCS/Codes/0/3/Q/Y/03QY4ZZ), [04QY0ZZ](https://www.icd10data.com/ICD10PCS/Codes/0/4/Q/Y/04QY0ZZ), [04QY3ZZ](https://www.icd10data.com/ICD10PCS/Codes/0/4/Q/Y/04QY3ZZ), [04QY4ZZ](https://www.icd10data.com/ICD10PCS/Codes/0/4/Q/Y/04QY4ZZ), [05QY0ZZ](https://www.icd10data.com/ICD10PCS/Codes/0/5/Q/Y/05QY0ZZ), [05QY3ZZ](https://www.icd10data.com/ICD10PCS/Codes/0/5/Q/Y/05QY3ZZ), [05QY4ZZ](https://www.icd10data.com/ICD10PCS/Codes/0/5/Q/Y/05QY4ZZ), [06QY0ZZ](https://www.icd10data.com/ICD10PCS/Codes/0/6/Q/Y/06QY0ZZ), [06QY3ZZ](https://www.icd10data.com/ICD10PCS/Codes/0/6/Q/Y/06QY3ZZ), [06QY4ZZ](https://www.icd10data.com/ICD10PCS/Codes/0/6/Q/Y/06QY4ZZ), [02QW0ZZ](https://www.icd10data.com/ICD10PCS/Codes/0/2/Q/W/02QW0ZZ), [02QW3ZZ](https://www.icd10data.com/ICD10PCS/Codes/0/2/Q/W/02QW3ZZ), [02QX4ZZ](https://www.icd10data.com/ICD10PCS/Codes/0/2/Q/X/02QX4ZZ), [03Q00ZZ](https://www.icd10data.com/ICD10PCS/Codes/0/3/Q/0/03Q00ZZ), [03Q03ZZ](https://www.icd10data.com/ICD10PCS/Codes/0/3/Q/0/03Q03ZZ), 03Q04ZZ, 03Q10ZZ, 03Q13ZZ, 03Q14ZZ, 03Q20ZZ, 03Q23ZZ, 03Q24ZZ, 03Q30ZZ, 03Q40ZZ, 03Q33ZZ, 03Q43ZZ, 03Q44ZZ, 03Q50ZZ, 03Q53ZZ, 03Q54ZZ, 03Q60ZZ, 03Q63ZZ, 03Q64ZZ, 03Q74ZZ 03Q70ZZ, 03Q73ZZ, 03Q80ZZ, 03Q83ZZ, 03Q84ZZ, 03Q90ZZ, 03Q93ZZ, 03Q94ZZ, 03QA0ZZ, 03QA3ZZ, 03QA4ZZ, 03QB0ZZ, 03QB3ZZ, 03QB4ZZ,03QC0ZZ, 03QC3ZZ, 03QC4ZZ,03QY0ZZ, 03QY3ZZ,03QY4ZZ,04Q00ZZ,  04Q03ZZ, 04QC0ZZ, 04QC3ZZ, 04Q04ZZ, 04QC4ZZ,04QD0ZZ, 04QD3ZZ,04QD4ZZ, 04QE0ZZ 04QE3ZZ, 04QE4ZZ, 04QF0ZZ, 04QF3ZZ, 04QF4ZZ, 04QH0ZZ, 04QH3ZZ, 04QH4ZZ,04QJ0ZZ, 04QJ3ZZ, 04QJ4ZZ,04QK0ZZ, 04QK3ZZ, 04QL0ZZ, 04QL3ZZ, 04QL4ZZ, 04QY0ZZ, 04QY3ZZ |
| Hemorrhage from vascular procedures | T82.838, T82.837 |
| Other artery and vein complications | T81.72XA, T81.719A |
| **Diaphragmatic paralysis** | J98.6 |
| **Reopen surgery** | 0W390ZZ, 0W3B0ZZ, 0W3C0ZZ, 0W3D0ZZ, 0W3Q0ZZ |
